# Dr. Glover on Scrofula

**Published:** 1847-04

**Authors:** 


### ART. XVI.

*On the Pathology and Treatment of Scrofula; being the Forthergillian Prize Essay for* 1846. By ROBERT MORTIMER GLOVER, M.D., &c. &c. —*London,* 1846. 8vo, pp. 315.

HAVING so recently reviewed the pathology and therapeutics of scrofula, in our notice of Mr. B. Phillips's most valuable publication, it is scarcely to be expected that we should go over the subject again. There are some points, however, in Dr. Glover's publication which merit notice. For several years, much attention has been given by the author (as we learn from the preface) to the subject of scrofula. In the year 1842, the Harveian Society of Edinburgh awarded him their medal for an essay on the physiological and medicinal properties of bromine, and on the analogies of the chlorine, bromine, and iodine group. In the spring of 1844, a dispensary was established in Newcastle, by Mr. Potter and him, with the assistance of some friends, for the treatment of scrofula, phthisis, and cutaneous affections and diseases of the joints, with the view of making an extensive trial of the therapeutical properties of some of the new remedies which have been proposed of late years in these affections, and especially with the view of trying the efficacy of the compounds of iodine. When the Medical Society of London announced their intention of awarding the Fothergillian gold medal for 1846, for the best essay on the pathology and treatment of scrofula, the author was induced to compete, and encouraged by the result, to publish. We have therefore a liber emeritus. Further, the work contains original investigations into the pathology of the disease, which were commenced in a right spirit, and under the guidance of sound principles. The microscope and chemical analysis have been used freely by him, the use of which he triumphantly defends:

"Many have drawn conclusions from the errors committed in the premature application of the resources and modes of research which we have alluded to; but it should be remembered that everything great and important must be bought at a price proportionate to its value. The failures which have taken place occurred in the infancy of chemical science, and before the existence of microscopic analysis as an art. Dr. Graves of Dublin has recently objected to the paucity of real results obtained by chemical analysis in the investigation of disease; but medicine has been two thousand years, according to the system of mere observation, in arriving at its present imperfect state, and the resources of chemistry have just begun to be applied, while every application made of them points at a vital part of our science, and tends to the solution of problems of the first magnitude. Henceforth morbid anatomy must not be confined to the department of the picture maker; but chemical and microscopical analysis should form the more important elements of this branch of pathology.

"The great difficulty to be contended with in medicine is the obscurity in which the connexion of observed facts is veiled. Hence the great value of anything approaching to experiment. The function of experiment must be distinguished from that of mere observation; experiment has in view the nature of the connexion between ascertained or observed facts, in order to test the constancy and essentiality of the relation; in other words, it is the bringing out of what Bacon terms prerogative facts. For instance, iodine is a remedy supposed capable of producing the absorption of scrofulous tumours. But a mere case in which a scrofulous person recovers under the use of iodine is of most moderate value: for patients have got rid of scrofula while submitted to every form of treatment, as proposed by generation after generation of so-called *practical men*, and remedy after remedy, thus used with apparent success, has fallen into oblivion. Suppose we were able to show that the use of iodine promotes not merely the flow of the urine, but also an increase of the solid contents in this fluid, and especially of the amount of urea? In other words, iodine excites the secondary digestion of the tissues; and as urea is the product of the albuminous tissues thus converted, and tubercle is composed chiefly of albumen, we learn that the connexion between the giving of the remedy and the absorption of a scrofulous tumour is not accidental, and thus we may derive a confidence which the blind empiricism so absurdly denominated *practical*, could never properly give. The sequel will prove that there is no wish here to undervalue observation of any kind; it is only intended to put prominently forward the great value of rationalism in medicine; and the reflections just written have been dictated by a review of the numerous failures in generalization presented by the history of the medical literature of scrofula;—failures which have chiefly arisen from too hastily grasping at the sequence and relationship of facts, without a sufficient consideration whether the connexion observed was essential and constant, or merely accidental." (pp. 5-8.)

After glancing at the different opinions regarding scrofula which has been brought forward, or have been current, Dr. Glover proceeds to discuss the pathology and treatment of scrofula, and first gives a description of scrofulous matter or tubercle. By this term, he wishes to designate a peculiar morbid formation, the product, as he considers, of a particular modification of the inflammatory process. This product is to be distinguished, firstly, from structures produced by ordinary (or what may with some liberty of speech be termed normal) inflammation; secondly, from various morbid growths inclining more or less to a parasitic character, and also from malignant structures; and thirdly, from various heterogeneous bodies of a totally different character, but which have sometimes been regarded as partaking more or less of the scrofulous deposit. Dr. Glover does not deny, that there may be cases in which scrofulous formations pass gradually into structures having characters different from those by which true scrofulous deposits are to be here described; this is only what we everywhere find in nature where absolute limits are unknown. As an example, may be noted those compound new formations, in which lymph of the usual structure, organized, is found to be intermingled with scrofulous matter presenting the characters of this formation when examined by the microscope.

"Tubercle, in the sense in which the term is here used, includes all scrofulous formations, whether in the lungs or in the lymphatic or lacteal glands, in the heart, the liver, the kidneys, the spleen, the brain, or spinal cord, the free surfaces of the mucous or serous membranes, the interstitial cellular membranes, the cellular tissue under the skin, the bones and periosteum, and, in short, in every conceivable tissue or organ; and we distinguish the scrofulous matter by its microscopic characters, with a certain not very definite chemical constitution, and a physical structure apparent to the eye, and which is well known. These characters, taken altogether, after the manner of the method of natural families in classifications of natural history, appear sufficient, as they can be described, to separate as accurately as can be done in any natural study, the scrofulous formations from all others." (p. 25.)

Dr. Glover next proceeds to notice the literary history of the external forms of tubercle, and then discusses the question as to its vascularity. He decides that the vascularity is not essential.

"The obliteration of the vessels of the tissue into which the scrofulous or tubercular matter is effused, may be carried to a greater or less extent; the inflammatory action produced by the presence of the tubercle on the surrounding tissues may, in some instances, cause obstruction of the vessels, or, in an earlier stage, increased vascularity; the envelopes which are formed around the tubercular mass may be more or less vascular, or totally devoid of vessels; but tubercle matter itself is *beyond the normal influence of the circulation;* and this fact is confirmed by the results of microscopic examination." (p. 37.)

The microscopic structure of tubercle, and the degree of organization it possesses, have given rise to much difference of opinion. Dr. Glover very succinctly reviews the opinions of the most recent investigators, including Canstatt, Vogel, Scharlau, Gluge, Kuhn, Gruby, Bennett of Edinburgh, Gulliver, and others. Dr. Glover's own investigations present results closely resembling those attained by Lebert and Dr. Bennett.

"Our own observations have been made with powers of four hundred, and six hundred and ten diameters, and on tubercles from the lungs, heart, spleen, renal capsules, kidneys and bladder, and on tuberculated mesenteric, bronchial, and cervical glands. The ordinary element of tubercle present in all the forms which we have examined, and scarcely different in one situation from what it is in another, is the granular corpuscle described by several writers. Many tubercular masses are composed almost wholly of this matter, which varies in size from about the bulk of a blood-globule to about, perhaps, the one 10,000th of an inch in diameter. These corpuscles are generally of a somewhat yellowish colour, and when magnified by the highest power which we have used (610 diameters) show occasionally spots in their substance which may possibly in some cases be nuclei. Mixed with these, which we believe to be in some instances altered cells, in other cases new formations, we have the following elements. 1st. Epithelial scales, variously altered, observed in lung tubercle, and which are shown in one of the drawings taken from a specimen of miliary tubercle. 2d. Fat-globules. 3d. Crystals of salts, of which some specimens are particularly exhibited in fig. 8 of the microscopic drawings. 4th. Portions of the destroyed tissues which sometimes assume singular shapes. 5th. Cells which also appear to belong to the old tissues; of these there is a specimen taken from tubercles of the heart in fig. 7, and the fig. 12 of Scherer's plate probably represents similar cells. 6th. Large granular and corpuscular masses of the most irregular forms." (pp. 49, 50.)

It thus appears, that there is no essential difference discernible in tubercles from various parts as to their microscopic constitution; while the products of normal inflammation and parasitic growths are readily distinguishable from tubercular deposits. In the latter, the formative power is deficient, and the cells which are formed either remain abortive, or granular corpuscles are developed instead of cells. In expressing this opinion, Dr. Glover only corroborates a similar doctrine enunciated by various pathologists.

*The chemical examination of scrofulous products.* Dr. Glover takes occasion to notice in detail all the more recent and trustworthy analyses of tubercular deposits, and then recounts a series of original analyses, twelve altogether, both of scrofulous deposit and pus. We subjoin the results of an analysis of 200 grains of crude mesenteric tubercle, after being freed from everything which had the appearance of ordinary tissue:

| | | |
|---|---|---|
| "The substance dried, furnished | . . . . . | 37·5 grs. |
| It was therefore composed of—Water | . . . . | 812·5 |
| Solids | . . . . | 187·5 |
| | | 1000·0 |

"The fats extracted by alcohol and ether, amounted to 7·2 grs., which burnt, gave 0·45 of saline residue; after the exhaustion by spirit of wine and water, there remained a residuum of 22·30 grs., and the half of the extractive matter removed in this manner, burnt, gave 0·25 of salts, 5 grs. of the protein residue, burnt for salts, gave 0·125 or 2·5 per cent. of ash.* No traces of casein or pyin were discovered. The proximate composition would then be:

| | | |
|---|---|---|
| Fats | . . . . . . . | 6·75 |
| Extractive matter sol. in spirits of wine and water, and loss | . . | 7·50 |
| Salts { Chlorides | . . . . . | 0·45 |
| Salts { Earthy salts (phosphates) | . . . . | 0·58 |
| Salts { Alkaline salts | . . . . . | 0·50 |
| Proteine residue | . . . . . | 21·72 |
| | | 37·50." (p. 73.) |

A mass of crude tubercle (Analysis 4) which weighed 500 grains, taken from a woman dead of phthisis, was selected on account of the completeness of the tuberculization. It was found to contain 786·4 parts water per thousand, and the proximate analysis was:

| | | |
|---|---|---|
| Fats and extractive substances by alcohol and ether, with salts | . | 18·25 |
| Spirituous extract, and loss | . . . . . | 4·00 |
| Watery extract and salts | . . . . . | 6·55 |
| Proteine residue and salts | . . . . . | 78·00 |
| | | 106·80 |

A mass of tubercle from the bronchial glands contained 25·8 per cent. of solid constituents, the composition of which was:

| | | |
|---|---|---|
| Fat acids | . . . . . . | 2·70 |
| Fatty body, non saponifiable—cholesterin (?) | . . . | 1·00 |
| Extractive matter, sol. in alcohol | . . . . | 1·30 |
| Extractive matter, by spirit of wine and hot water | . . | 4·10 |
| Insoluble proteine, residue, and loss | . . . . | 15·00 |
| Salts | . . . . . . . | 1·70 |
| | | 25·80 |

A tuberculated cervical gland of an ox, which contained cretaceous fragments, weighed 25 grains when dried, of which 3·85 grains were phosphate of lime. A portion of deposit from the upper part of a tuberculous lung, "exhibiting what appeared to be a partial change to the cretaceous substance," was found to consist of:

| | | |
|---|---|---|
| Animal matter | . . . . . , | 24·80 |
| Salts | . . . . . . . | 9·00 |
| | | 33·80 |
| Chlorides, with a trace of alkaline salts | . . . | 1·22 |
| Salts soluble in water, as above described | . . . | 1·90 |
| Phosphate of lime, a trace, and loss | . . . . | 0·48 |
| | | 9·00 |

* Three grs. were tested for casein with acids; it dissolved readily by the aid of heat in acetic muriatic, and strong sulphuric acids.

With regard to the results of the analyses of pus, Dr. Glover observes that scrofulous pus appears to differ from ordinary pus, chiefly in the fluid part being thinner and mixed with albuminous granules, proceeding from a decomposition of scrofulous or tuberculous matter. The pus-globules appear also, as stated by Mr. Gulliver, to be fewer and less distinct than those of healthy pus. He found them also more irregular in their form.

*State of the blood in scrofula.* Dr. Glover here again reviews the facts and opinions promulgated by his predecessors, and then details a series of original analyses after the method of Andral and Gavarret, of the blood of scrofulous persons, namely 11 males and 17 females. The means resulting from the analysis of the blood of males was found to be—solids 208·05, fibrin 3·132, solids of serum 87·60, globules 117·32. We subjoin Dr. Glover's reflections and deductions:

"On the whole, therefore, it may be stated, as the result of these analyses, that in scrofula we have an increase in the solids of the serum, and a diminution of blood-globules, which is very nearly the alteration that has been long suspected to exist. As far as the analyses go, the fats are not deficient in the blood, and however opposed this may be to certain hypothetical notions, with regard to the supposed *modus operandi* of cod-liver oil, it is altogether in harmony with the results of the analysis of tubercle which we have made. The experiments of Ascherson have been supposed to prove the capability of fats introduced into the blood, to work up mechanically the excess of serous matter into blood-globules, and so counteract the tendency to the expulsion of this serous matter, and its excretion into the tissues under the form of tubercle. But we find a large portion of fats in tubercles; in one analysis, conducted on a sufficient scale, almost one fourth of the tubercular matter was found composed of fats. And this result is not much in discordance with other analyses, and with analyses by other persons. If then the diseased matter be expelled by a supposed effort of the vis medicatrix, it would appear as if the sanative materials were also expelled. But this theory depending upon the tendency of an albuminous liquid to form spherical globules in contact with oil, is altogether too mechanical, and vitiates itself by giving a too easy explanation of great difficulties." (p. 115.)

The condition of the bile, lymph, and chyle, in scrofula, are not elucidated by any original researches instituted by Dr. Glover; there is, however, as usual, a succinct summary of the current facts and opinions, but which are meagre and unsatisfactory. The condition of the urine in scrofula was investigated in nine cases. Case 1.—A boy, aged 15, with swelling and suppuration of the cervical glands. The patient took syrup of iodide of iron (drachm dose thrice daily) and used frictions of hydriodate of potass ointment on the tumours. After using them six weeks, iodine was found present in the urine in large quantity, and also traces of iron; and in fact in all the other analyses of the urine of patients using iodine or its preparations, that substance abounded in the urine. Otherwise, the analyses do not show any particular change in the composition of the urine of scrofulous patients.

The scrofulous diathesis and its external signs occupy a chapter of Dr. Glover's work. On this head we do not find much novelty. In 126 cases which Dr. Glover examined, 86 had light hair and complexion, and the remaining 40 were dark. But the inmates of three workhouses situate in the districts which furnished the cases, bore nearly the same proportion of light and dark complexions, there being 97 of the former and 47

of the latter amongst 144 individuals. So that in point of fact the light-haired scrofulous cases were in the greater proportion, because the light-haired race was in the greater proportion amongst the general population.

Scrofulous diseases exist to a considerable extent amongst brutes, and this circumstance has led Dr. Glover to devote a chapter of his work to the comparative pathology of scrofula. It is wholly, however, a summary of facts and opinions already published.

The question as to the identity of scrofulous and tubercular diseases is maintained in the affirmative by Dr. Glover, and we think in the most satisfactory manner. As we have already gone over this question in our review of Mr. Phillips's work, and as we think by far the larger proportion of practitioners treat the question as already settled in accordance with the views there expressed, we think it unnecessary to revert to it. Those, however, who wish for a concise statement of the argument, will find it well made by Dr. Glover.

As to the essential nature of scrofula—that is to say, the actual diseased process, Dr. Glover defines it to consist in "a peculiar modification of inflammation, whereby the usual, or, as they may be termed, the normal products of this process are not evolved, but instead of them other materials incapable of passing into the regular cell-forms, and which constitute the substance already described under the name of scrofulous or tubercular matter. The peculiarity of this formation, and the continuance of the scrofulous diathesis, are the causes of the characters assumed by the various after-processes which result from the existence of tubercles." The word "peculiar" might well be left out, but we have not space to go over the lucid theoretical discussion Dr. Glover enters upon in support of his views: we subjoin the following, however, as an illustration of his method, and the record of an interesting fact:

"Among the arguments usually advanced in favour of the production of tubercles by inflammation, is the formation of them by artificial means. We have produced them in this way in the rabbit and dog, chiefly in order to examine them by the microscope. The mode of experimenting adopted was by incising the trachea, and injecting a quantity of mercury downward into the lungs. The appearance of the bodies which resulted, the animal being killed at a period of from one to two months after the operation, was not externally unlike that of tubercle; little round whitish masses, more or less agglomerated; and each nodule with a globule of mercury in its centre, around which the exuded matter was formed. Pus existed in some parts around the artificial tubercles; but on examining the broken-up bodies by the microscope, although the structure of portions did not appear very different from that of the irregular granules and corpuscles of tubercle, yet the exudation-corpuscles were tolerably numerous, and those towards the edges of the exudation fully formed. Numerous nucleated cells also were found mixed with the mass; in short the formation more nearly resembled an ordinary inflammatory product, such as we find in pulmonary hepatization." (p. 195.)

In this discussion of the etiology of Scrofula, we again find our views corroborated by Dr. Glover. He states truly and with great candour, that Mr. Phillips's work contains by far the best account of the etiology of scrofula ever yet given to the world. We are, however, bound to say, that highly as we have estimated Mr. Phillips's work, we think Dr. Glover has discussed certain points in the etiology with even more philosopical precision than Mr. Phillips: we refer more particularly to the hereditary transmission of scrofula. "Surely," he observes, "the *fact* that in many instances scrofula is hereditarily transmitted does not now depend upon the researches or opinions of any one individual!" The following is perfectly sound, and is followed by illustrative cases:

"That family peculiarities may pass over one generation and appear in another, is a fact too well established to be denied. Now, as the transmission of the actual disease is admitted to be a very rare event; and as in the various questions connected with this part of the subject, the *real question* is about the diathesis or predisposition, we cannot limit the investigation merely to the parents and offspring. The existence of tuberculous affections also in several of the children, where no adequate occasional causes can be discovered, is, even where the parents are free, a suspicious circumstance. To limit the investigation also to external tubercle, would be as unphilosophical on the view, advocated here, of the ultimate nature of the pathological connexion between the various forms of tubercle. How, upon this principle, could we pronounce that the disease was not hereditary, where we found that the parent of a scrofulous child had died of phthisis?" (pp. 203-4.)

*The treatment of scrofula* is discussed by Dr. Glover under the two heads *hygienic* and *medicinal*. As to the first, we have nothing new; good nursing in infancy, a good physical education in youth, care in the selection of a matrimonial partner at manhood,—these are the principal views of the hygiene of scrofula. With regard to the medicinal, we subjoin the following words:

"Where the tongue is furred, or spotted with reddish maculæ, the pulse quick and irritable, and the bowels constipated, a state very common at the commencement of scrofulous tuberculization, benefit will be often derived from the use of hydrarg. c. creta, in a dose of three or four grains twice a day, along with seven or eight grains of magnesia, combined sometimes with rhubarb. In some cases we have seen enlargements of the glands disappear under this treatment, without the use of other remedies." (p. 240.)

We agree with Dr. Glover in his statement, that the total proscription of mercury is as unwarrantable as the exclusive use of iodine, or cod-liver oil.

"In truth considerable analogy exists between the action of mercury and that of iodine: the compounds of both substances are powerfully irritant in large doses, tonic and alterative in small doses, and given so as to act constitutionally, appear to have the power of greatly accelerating and increasing the destructive digestion of the tissues; and both, when pushed to excess, give rise to dangerous constitutional effects connected with a peculiar erethism. Mercury is undoubtedly the more powerful remedy of the two. But in our practice we are not deterred by chimeras from the use of mercury in scrofula. Indeed, the most potent remedy which we possess for effecting the removal of a scrofulous tumour, is in our opinion the sub-iodide of mercury, a substance whose powers of salivation have been strangely doubted; but the properties of compounds follow the rule of the base, rather than that of the electro-negative element; and the sub-iodide is a true mercurial compound." (p. 241.)

With regard to the compounds of iodine, bromine, and chlorine, it is the opinion of Dr. Glover, founded on experiments, that they are active in proportion to their solubility and facility of decomposition. Tonics are admissible in every stage; Dr. Glover combines calumba with the saccharine carbonate of iron. The action of cod-liver oil is that of a tonic, due he thinks to the resinous principle it contains, and to its power in stimulating the development of animal heat, (?) and to the fact that it occasionally acts as an aperient and diuretic.

*Particular remedies for scrofula.* Dr. Glover discusses these under seven heads, digitalis and walnut leaves heading the list, and electricity completing it. We shall only make some excerpta. Of the three non-metallic elementary bodies, which have a certain analogy of therapeutic action, as well as of chemical relation, Dr. Glover observes, that it is probable chlorine is the most potent, bromine the next, and iodine the next, judging from comparative experiments on animals, and in accordance with the great law which makes strict relation subsist between the chemical, and the physiological and medicinal properties of bodies. Bromine, in the proportion of eight or twelve minims to a pint or half a pint of water, makes an elegant lotion. Dr. Glover prefers in ordinary cases to give iodine in the form of the compound tincture of the London Pharmacopœia, beginning with 25 drops thrice a day, and increasing the dose gradually to 30 or 40 drops. Its effect is to act as a general tonic, improve the appetite and increase the quantity of solids and urea in the urine, as well as the quantity of urine itself.

With regard to the compounds of these elementary bodies, Dr. Glover is of opinion, that the chloride of potassium might be advantageously substituted for the iodide, and in a certain class of cases might be also substituted. The bromide excites less nausea than the iodide, and in a certain class of cases might be also substituted. The bromide and iodide of barium have the same physiological properties as the chloride; Dr. Glover details a case confirmatory of a statement in a recent number of this Journal, that the iodide of barium is apt to act too energetically on the uterine system. We have observed, that the iodide of potassium has this effect occasionally, and that menstruation has apparently been induced by it after a long interval of inaction.

Dr. Glover thinks the value of sea-air and sea-water as remedies in scrofula, may be in some degree dependent upon the chlorine in the former and the chlorides in the latter. To ascertain the presence of chlorine in the atmosphere, Dr. Glover forced a quantity of air through a vessel containing a solution of nitrate of silver for several minutes, the result, was (the atmosphere being very calm, and the slight wind not directly from the sea, at about thirty paces from the sea-shore) a distinct milkiness of the water, although there was no precipitate. In England, the mineral waters of Shap and Shotley may be useful in scrofula on account of the large quantity of chlorides they contain. Cod-liver oil is recommended in cachectic cases. Dr. Glover says, "we have weighed (by 'we' meaning *we* suppose 'I') phthisical patients and others, taking it, from time to time, and have found them sometimes grow stouter, even when the disease was unchecked."

An appendix of cases closes the work.

We feel we ought to congratulate Dr. Glover on the able manner in which he has handled his subject. We cannot give his book higher praise than to say that it may fairly bear comparison with that of Mr. Phillips, although totally different in its method. The two volumes are complementary of each other, and both claim the attentive study of the profession.

